# O Eletrocardiograma como Janela para a Patologia Atrial: Percepções de uma Grande Coorte Brasileira sobre Cardiomiopatia Atrial e Acidente Vascular Cerebral

**DOI:** 10.36660/abc.20260270

**Published:** 2026-06-24

**Authors:** M. Julia Machline-Carrion

**Affiliations:** 1 Nexus Research Partners Londres Reino Unido Nexus Research Partners, Londres – Reino Unido

**Keywords:** Fibrilação Atrial, Saúde Populacional, Prevenção de AVC

O acidente vascular cerebral (AVC) continua sendo uma das principais causas de morte e incapacidade em todo o mundo, com eventos isquêmicos responsáveis por aproximadamente 87% dos casos. Apesar da investigação etiológica exaustiva, até 25% dos AVCs isquêmicos permanecem sem explicação — o chamado AVC criptogênico — uma designação que acarreta implicações significativas para a prevenção secundária, dada uma taxa média de recorrência de 3–6% ao ano.^1,2^ A fibrilação atrial (FA) tem sido, por muito tempo, o principal fator de risco cardioembólico nesse contexto; no entanto, evidências crescentes sugerem que anormalidades atriais estruturais e funcionais — coletivamente englobadas pelo conceito de cardiomiopatia atrial (CA) — podem impulsionar eventos tromboembólicos por meio de uma via independente, mesmo na ausência de arritmia manifesta.^3^ A declaração de consenso atualizada de 2024 da EHRA, HRS, APHRS e LAHRS consolida um sistema de estadiamento para CA integrando biomarcadores, geometria atrial e parâmetros eletrofisiológicos, estabelecendo firmemente a CA como uma entidade patológica distinta da — embora intimamente associada à — FA.^4^ Nesse contexto, o eletrocardiograma (ECG) de 12 derivações — simples, barato e universalmente disponível — oferece uma abordagem prática para a detecção de CA por meio de dois marcadores estabelecidos: duração prolongada da onda P (>120 ms) e força terminal da onda P elevada na derivação V1 (PTFV1 >4.000 μV·ms). Estudos anteriores do *Cardiovascular Health Study* (CHS), do *Multi-Ethnic Study of Atherosclerosis* (MESA) e da coorte *Atherosclerosis Risk in Communities* (ARIC) demonstraram que esses marcadores predizem independentemente o AVC isquêmico;^5–7^ no entanto, sua relevância em populações latino-americanas, com um perfil cardiovascular e sociodemográfico distinto, permaneceu uma importante lacuna de evidências.

Nesta edição dos Arquivos Brasileiros de Cardiologia, o estudo de Lêu et al.^8^ aborda essa lacuna com notável rigor. Utilizando o conjunto de dados CODE-BH — um subconjunto da coorte *Clinical Outcomes in Digital Electrocardiography* (CODE), um dos maiores bancos de dados de ECG digital anotado do mundo^9^ — os autores reuniram uma coorte retrospectiva de 245.588 pacientes com idade ≥40 anos em ritmo sinusal, que realizaram ECGs em Belo Horizonte entre 2006 e 2018. Os dados eletrocardiográficos foram vinculados probabilisticamente aos bancos de dados do Sistema Único de Saúde (SUS) — o Sistema de Informações sobre Mortalidade (SIM) e o Sistema de Informações Hospitalares (SIH) — permitindo a identificação de óbitos e internações por AVC, mortalidade cardiovascular, FA incidente e síndrome coronariana aguda durante um acompanhamento médio de 3,5 anos. Após ajuste incremental para idade, sexo, fatores de risco cardiovascular e hipertrofia ventricular esquerda, tanto a duração prolongada da onda P (HR 1,24; IC 95% 1,12–1,36) quanto a PTFV1 elevada (HR 1,20; IC 95% 1,05–1,38) foram associadas independentemente ao desfecho composto de morte ou hospitalização por AVC. A duração prolongada da onda P também foi associada à mortalidade cardiovascular e à incidência de FA, enquanto a PTFV1 elevada foi associada à mortalidade por todas as causas e à mortalidade cardiovascular.

Diversos pontos fortes metodológicos merecem reconhecimento. O tamanho da amostra — o maior entre estudos comparáveis até o momento — confere considerável poder estatístico, compensando efetivamente o período médio de acompanhamento relativamente curto. A plataforma CODE já gerou contribuições de influência internacional, incluindo trabalhos pioneiros sobre interpretação de ECG baseada em redes neurais profundas^10^ e idade biológica derivada do ECG como preditor de mortalidade,^11^ posicionando esta coorte como uma plataforma de relevância científica global. A adesão às diretrizes de relato STROBE fortalece ainda mais a transparência metodológica, e o uso da análise automatizada de ECG de Glasgow garante a reprodutibilidade em todos os contextos de atenção primária de onde a coorte foi extraída. É importante destacar que este estudo constitui a primeira investigação de marcadores de CA baseados em ECG e desfechos cardiovasculares adversos em uma população brasileira, conferindo-lhe particular relevância para a prática cardiológica regional.

Como em todos os estudos que utilizam bases de dados administrativas de saúde, certas características do desenho do estudo também definem oportunidades para pesquisas futuras. O uso de códigos da CID-10 do SIM e do SIH para identificar eventos de AVC — abrangendo diagnósticos isquêmicos, hemorrágicos e cerebrovasculares não especificados — reflete uma característica inerente à metodologia de coorte eletrônica em larga escala e abre uma via de pesquisa promissora: estudos futuros que relacionem esses marcadores de ECG a subtipos de AVC confirmados por neuroimagem e a sistemas de classificação etiológica validados, como o TOAST^12^ ou os critérios ESUS,^13^ permitiriam aos pesquisadores determinar se a associação com a CA é impulsionada especificamente por eventos cardioembólicos — como a plausibilidade biológica da hipótese preveria — e quantificar a contribuição relativa da CA para cada mecanismo de AVC. Da mesma forma, o contexto de atenção primária da coorte, embora limite a generalização para populações clínicas de maior risco, ilustra precisamente a amplitude e a eficiência com que o rastreamento de CA baseado em ECG poderia ser implementado em nível populacional. A subestimação da incidência de FA devido a registros de ECG em um único momento destaca o potencial de estratégias de monitoramento contínuo — incluindo gravadores de eventos implantáveis e ECG ambulatorial aprimorado por IA — para refinar a estratificação de risco quando combinadas com marcadores da onda P em futuros protocolos prospectivos.

O panorama terapêutico para a prevenção de AVC relacionado à anticoagulação permanece uma fronteira ativa. O estudo ARCADIA, que randomizou pacientes com AVC criptogênico e evidência de cardiopatia atrial para apixabana versus aspirina, não demonstrou redução significativa na recorrência de AVC com anticoagulação na ausência de FA documentada,^14^ ressaltando que marcadores atriais estruturais isoladamente podem ser insuficientes para orientar as decisões de anticoagulação na prática atual e que a identificação de fenótipos específicos de alto risco de anticoagulação permanece uma necessidade crítica não atendida. Uma revisão sistemática e meta-análise recentes confirmam que a duração da onda P basal é significativamente prolongada em sobreviventes de AVC criptogênico que posteriormente desenvolvem FA, reforçando o ECG como uma ferramenta prática de triagem para selecionar pacientes que podem se beneficiar de monitoramento cardíaco prolongado.^15^ As Diretrizes da ESC de 2024 sobre FA, que introduziram a estrutura AF-CARE centrada no paciente, reconhecem explicitamente o papel crescente da avaliação do substrato atrial na prevenção de AVC, fornecendo um contexto regulatório oportuno no qual os presentes achados são particularmente relevantes.^16^

Em resumo, o estudo^8^ representa uma contribuição significativa para a compreensão crescente da CA como um fator de risco independente para AVC e mortalidade cardiovascular. Seus achados corroboram o valor prognóstico de parâmetros eletrocardiográficos amplamente disponíveis e reforçam a hipótese de que a doença do substrato atrial pode representar uma via distinta e passível de intervenção na prevenção do AVC cardioembólico. O estudo também abre caminho para futuras investigações prospectivas capazes de alcançar a resolução etiológica e a granularidade clínica necessárias para traduzir essas associações em estratégias de prevenção eficazes — e, em última análise, reduzir a carga de AVC criptogênico na população brasileira e latino-americana em geral.
