# Supplementary material for: O Eletrocardiograma como Janela para a Patologia Atrial: Percepções de uma Grande Coorte Brasileira sobre Cardiomiopatia Atrial e Acidente Vascular Cerebral
Source: Arq Bras Cardiol. 2026 Jun 24;123(3):e20260270. [Article in Portuguese] doi: 10.36660/abc.20260270 (PMC13398831; doi:10.36660/abc.20260270)
Supplement: Translation

# Translation: The Electrocardiogram as a Window into Atrial Pathology: Insights from a Large Brazilian Cohort on Atrial Cardiomyopathy and Stroke

**DOI:** 10.36660/abc.20260270i

**Authors:** M. Julia Machline-Carrion

**Affiliations:** 1 London United Kingdom Nexus Research Partners, London – United Kingdom

**Keywords:** Atrial Fibrillation, Data Insights, Population Health, Stroke Prevention

Stroke remains one of the foremost causes of death and disability globally, with ischemic events accounting for approximately 87% of cases. Despite exhaustive etiological investigation, up to 25% of ischemic strokes remain unexplained — the so-called cryptogenic stroke — a designation that carries significant implications for secondary prevention, given an average recurrence rate of 3–6% per year.^1,2^ Atrial fibrillation (AF) has long been the dominant cardioembolic risk factor in this setting; however, mounting evidence suggests that structural and functional atrial abnormalities — collectively encompassed by the concept of atrial cardiomyopathy (AC) — may drive thromboembolic events through an independent pathway, even in the absence of overt arrhythmia.^3^ The 2024 updated consensus statement from EHRA, HRS, APHRS, and LAHRS consolidates a staging system for AC integrating biomarkers, atrial geometry, and electrophysiological parameters, firmly establishing AC as a pathological entity distinct from — though intimately associated with — AF.^4^ In this context, the 12-lead electrocardiogram (ECG) — simple, inexpensive, and universally available — offers a practical approach to AC detection through two established markers: prolonged P-wave duration (>120 ms) and elevated P-wave terminal force in lead V1 (PTFV1 >4,000 μV·ms). Prior studies from the Cardiovascular Health Study (CHS), the Multi-Ethnic Study of Atherosclerosis (MESA), and the Atherosclerosis Risk in Communities (ARIC) cohort demonstrated that these markers independently predict ischemic stroke;^5–7^ yet their relevance in Latin American populations, with a distinct cardiovascular and sociodemographic profile, had remained an important evidence gap.

In this issue of *Arquivos Brasileiros de Cardiologia*, the study by Lêu et al.^8^ addresses this gap with notable rigour. Drawing upon the CODE-BH dataset — a subset of the Clinical Outcomes in Digital Electrocardiography (CODE) cohort, one of the world's largest annotated digital ECG databases^9^ — the authors assembled a retrospective cohort of 245,588 patients aged ≥40 years in sinus rhythm, who underwent ECGs in Belo Horizonte between 2006 and 2018. Electrocardiographic data were probabilistically linked to the Brazilian Unified Health System (SUS) databases — the Mortality Information System (SIM) and the Hospital Information System (SIH) — enabling ascertainment of stroke-related deaths and hospitalizations, cardiovascular mortality, incident AF, and acute coronary syndrome over a mean follow-up of 3.5 years. After incremental adjustment for age, sex, cardiovascular risk factors, and left ventricular hypertrophy, both prolonged P-wave duration (HR 1.24; 95% CI 1.12–1.36) and elevated PTFV1 (HR 1.20; 95% CI 1.05–1.38) were independently associated with the composite endpoint of stroke-related death or hospitalization. Prolonged P-wave duration was further associated with cardiovascular mortality and incident AF, whilst elevated PTFV1 was linked to all-cause and cardiovascular mortality.

Several methodological strengths deserve recognition. The sample size — the largest among comparable studies to date — confers considerable statistical power, effectively offsetting the relatively short mean follow-up. The CODE platform has already yielded internationally influential contributions, including pioneering work on deep neural network-based ECG interpretation^10^ and ECG-derived biological age as a mortality predictor,^11^ positioning this cohort as a platform of global scientific relevance. Adherence to STROBE reporting guidelines further strengthens methodological transparency, and the use of automated Glasgow ECG analysis ensures reproducibility across the primary care settings from which the cohort was drawn. Importantly, this study constitutes the first investigation of ECG-based AC markers and adverse cardiovascular outcomes in a Brazilian population, lending it particular significance for regional cardiology practice.

As with all studies leveraging administrative health databases, certain design characteristics also define opportunities for future research. The use of ICD-10 codes from SIM and SIH to identify stroke events — encompassing ischemic, hemorrhagic, and unspecified cerebrovascular diagnoses — reflects an inherent feature of large-scale electronic cohort methodology, and opens a compelling research avenue: future studies linking these ECG markers to neuroimaging-confirmed stroke subtypes and validated etiological classification systems, such as TOAST^12^ or the ESUS criteria,^13^ would allow investigators to determine whether the association with AC is driven specifically by cardioembolic events — as the biological plausibility of the hypothesis would predict — and to quantify the relative contribution of AC to each stroke mechanism. Similarly, the primary care setting of the cohort, whilst limiting generalizability to higher-risk clinical populations, illustrates precisely how widely and efficiently ECG-based AC screening could be deployed at a population level. The underestimation of incident AF due to single-timepoint ECG recordings highlights the potential of continuous monitoring strategies — including implantable loop recorders and AI-enhanced ambulatory ECG — to refine risk stratification when combined with P-wave markers in future prospective protocols.

The therapeutic landscape for AC-related stroke prevention remains an active frontier. The ARCADIA trial, which randomized patients with cryptogenic stroke and evidence of atrial cardiopathy to apixaban versus aspirin, did not demonstrate a significant reduction in recurrent stroke with anticoagulation in the absence of documented AF,^14^ underscoring that structural atrial markers alone may be insufficient to guide anticoagulation decisions in current practice, and that identification of specific high-risk AC phenotypes remains a critical unmet need. A recent systematic review and meta-analysis confirms that baseline P-wave duration is significantly prolonged in cryptogenic stroke survivors who subsequently develop AF, reinforcing the ECG as a practical triage tool for selecting patients who may benefit from extended cardiac monitoring.^15^ The 2024 ESC Guidelines on AF, which introduced the patient-centered AF-CARE framework, explicitly acknowledge the growing role of atrial substrate assessment in stroke prevention, providing a timely regulatory context within which the present findings are particularly resonant.^16^

In summary, the study^8^ constitutes a meaningful contribution to the evolving understanding of AC as an independent risk factor for stroke and cardiovascular mortality. Its findings substantiate the prognostic value of widely available ECG parameters and reinforce the hypothesis that atrial substrate disease may represent a distinct, targetable pathway in cardioembolic stroke prevention. The study also paves the way for future prospective investigations capable of achieving the etiological resolution and clinical granularity needed to translate these associations into actionable prevention strategies — and ultimately to reduce the burden of cryptogenic stroke in the Brazilian and broader Latin American population.
